# Advances of Optofluidic Microcavities for Microlasers and Biosensors

**DOI:** 10.3390/mi9030122

**Published:** 2018-03-09

**Authors:** Zhiqing Feng, Lan Bai

**Affiliations:** 1 College of Physics and Materials Engineering, Dalian Nationalities University, Dalian 116600, China; fzq@dlnu.edu.cn; 2 College of Mechanical and Electronic Engineering, Dalian Nationalities University, Dalian 116600, China

**Keywords:** microcavities, optofluidic dye lasers, whispering gallery modes, biosensors

## Abstract

Optofluidic microcavities with high Q factor have made rapid progress in recent years by using various micro-structures. On one hand, they are applied to microfluidic lasers with low excitation thresholds. On the other hand, they inspire the innovation of new biosensing devices with excellent performance. In this article, the recent advances in the microlaser research and the biochemical sensing field will be reviewed. The former will be categorized based on the structures of optical resonant cavities such as the Fabry–Pérot cavity and whispering gallery mode, and the latter will be classified based on the working principles into active sensors and passive sensors. Moreover, the difficulty of single-chip integration and recent endeavors will be briefly discussed.

## 1. Introduction

In recent years, optofluidic microcavities have been developed, becoming a key element of microfluidic platforms. Many kinds of microcavities with high Q value and small mode volume have been obtained by using microfabrication technology [1,2,3,4,5,6,7,8,9] thanks to their excellent light confinement for a long time in a small volume. They enjoy a significant enhancement of light-matter interaction and narrow resonance linewidth, making them favorable for optofluidic microlasers and biochemical sensing applications.

**Figure 1:**
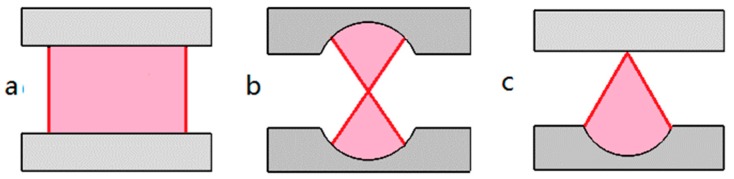
Three configurations of Fabry–Pérot (FP) microcavity, (**a**) plane-plane mirror type (PPFP). (**b**) concave-concave mirror type (CCFP). (**c**) plane-concave mirror type (PCFP).

**Figure 2:**
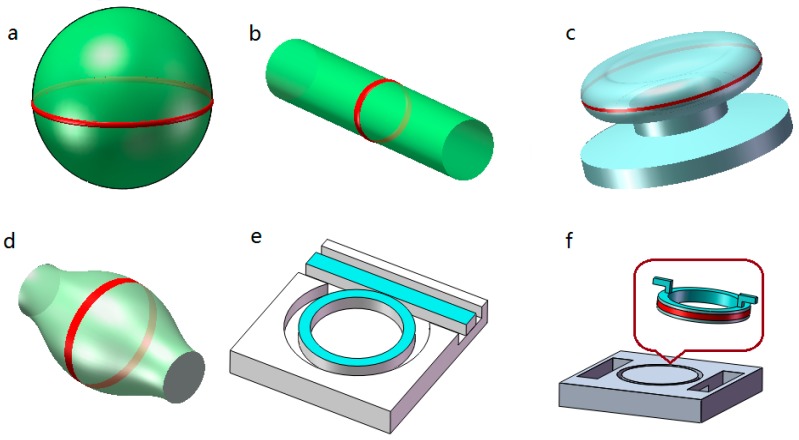
Classical configurations of whispering gallery mode (WGM)-based microcavities. (**a**) Microsphere; (**b**) cylindrical ring; (**c**) microdisk or microtoroid; (**d**) microbottle; (**e**) monolithic solid core microring; (**f**) monolithic liquid core microring.

Depending on the light confinement mechanism, the microcavities are generally divided into two categories: Fabry–Pérot (FP) cavities and whispering gallery mode (WGMs) cavities. The materials can be optical fibers, microcapillaries, polymers, silicon or glass substrates. As illuminated in Figure 1, there are usually several common FP microcavities, as defined by their geometric shapes: plane-plane mirror type (PPFP), concave-concave mirror type (CCFP) and plane-concave mirror type (PCFP). Figure 2 shows more kinds of WGM microcavities, including microring, microdisk, microtoroid, microsphere, microbubble and microbottle.

Optofluidic dye lasers are formed by integrating microcavities and gain medium into proper microfluidic circuits or devices. Lots of gain media have been used, such as dyes, quantum dots, rare earth ions, labeled-DNA, fluorescent proteins, chlorophyll solutions, etc. As miniaturized light sources, the optofluidic dye lasers have the merits of low threshold and high Q factor. In addition, they have made significant advances in other aspects such as full bio-compatibility, mode selecting between single and multi-mode, lasing wavelength tunability, and so on. As biochemical sensing elements, the optofluidic lasers usually obtain much higher sensitivity than traditional detecting techniques [10,11,12,13,14,15,16,17]. For sensing applications, they are also described as active resonator sensors.

Optofluidic microcavities can also be worked as passive resonator sensors without gain medium. Recent research has mainly focused on the WGM-based resonators [18,19,20,21,22,23,24,25,26,27]. The sensing ability of WGM resonators is characterized by the figure of merit using the Q-factor value to mode volume ratio (Q/V). The sensitivity can be improved by increasing the Q value or decreasing the resonators’ mode volume. Through ultrafine laser processing, many microscale resonators have been fabricated, and their detection limits have been successfully reduced down to several kDa molecular weight for single particles [19,20,21,22,23,24,25].

This article is not the first to review the topic of optofluidic microcavities. In 2010, Y. Chen gave an in-depth review on the physical theory and the development status of optofluidic microcavities [28]. However, the research of optofluidic microcavities has made substantial progress since then, and many inspiring studies have emerged. It is now a good time to update the latest research progress of optofluidic microcavities in two application areas: optofluidic dye lasers and microcavity-based biosensors.

## 2. Optofluidic Microcavities for Dye Lasers

### 2.1. Fabry–Pérot Cavity Dye Lasers

**Figure 3:**
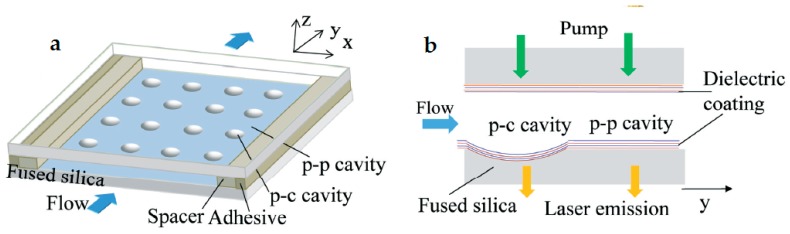
(**a**) Schematic of the optofluidic laser array based on the PCFP and PPFP cavities. (**b**) Details of the experimental setup employing both the PCFP and the PPFP cavity on the same fused silica chip [29].

The FP cavities are easy to fabricate. For example, optical fiber end faces or glass slides can be used to constitute the PPFP cavity; microscale concaves (or concave arrays) made by laser machining on planar substrates can form the PCFP cavity [29,30,31,32,33,34,35]. Figure 3 shows the PCFP cavity array structure made by Wang [29]. By depositing the Bragg reflection dielectric layers, the Q value was enhanced to 5.6 × 10^5^. When the cavity length was 31 μm, the laser threshold was lowered to 0.09 μJ·mm^−2^. When the cavity length was shortened to 8 μm, the excitation threshold was increased to 0.5 μJ·mm^−2^, and single mode lasing was observed at 599 nm. The Lahoz group reported another simple design of a PPFP dye laser [31] which could be excited by a low-power continuous-wave (CW) laser diode with the threshold of 1.3 μJ·mm^−2^. As a sensor, it could be operated in laser mode or fluorescence mode by changing the excitation laser intensity.

**Figure 4:**
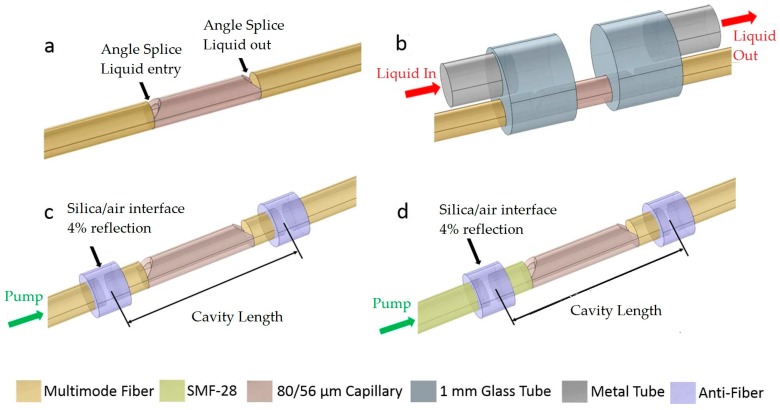
Device schemes of the all-fiber high-repetition-rate microfluidic dye lasers. (**a**) Angle-cleaved capillary spliced to conventional fibers, allowing for liquid flow; (**b**) a whole device, including the pressure cells formed by glass tubes and their connection to liquid reservoirs via metal tubes; (**c**) multimode laser cavity, including air gaps for the feedback via Fresnel reflection; (**d**) few-mode laser cavity with similar air gaps but with a small-core fiber (SMF-28) in one side to provide modal filtering. Anti-fiber is the capillary (inner diameter 128 µm) used to generate the air gaps [33].

Gerosa [33] constructed all-fiber high-repetition-rate microfluidic dye lasers by welding the optical fibers and the capillary tubes. The excitation threshold was about 1 μJ by using 532-nm, 300-ps 1-kHz pulse laser. The structure is illuminated in Figure 4. Some key features of the FP cavity dye lasers are listed in Table 1.

### 2.2. WGM Dye Lasers

The WGM dye lasers are obtained by the combination of a liquid or solid state gain medium and WGM microresonators. If the medium around the microresonator has positive optical gain, the evanescent wave of the WGMs would interact with the medium to generate WGM laser emission. Various forms of resonators have been demonstrated, such as microring, microsphere, microbubble, microdisc, microtoroid, and microbottle [36,37,38,39,40,41,42,43,44,45,46,47,48,49,50,51,52,53,54,55,56,57,58,59,60,61,62]. Cylindrical and planar microring are more popular due to their simple configurations which confine the photon propagation in the quasi-two-dimensional space. Recently, a solid or hollow microbottle based on microcylinder or microcapillary structures has been proposed for the WGM lasers. The advantage of the microbottle structure is that it has multiple non-degenerated modes along the axis of the revolution, which are convenient for modes selection. Single-mode lasing can be realized by spatial pump engineering [42,43,44]. Here, we list in Table 2 the main features of the recently proposed WGM dye lasers.

In the WGM lasers, the carrier utilizes the hollow microstructured fibers, the microcapillaries or the planar microrings on chips, and the gain medium liquid is filled in or flowed through. In some other designs, the dye-doped polymer is coated on the inner or outer wall of resonator to form the microring resonator dye lasers using the side or axial pumping. The cavity length of cylindrical resonators can be further reduced by tapering.

**Figure 5:**
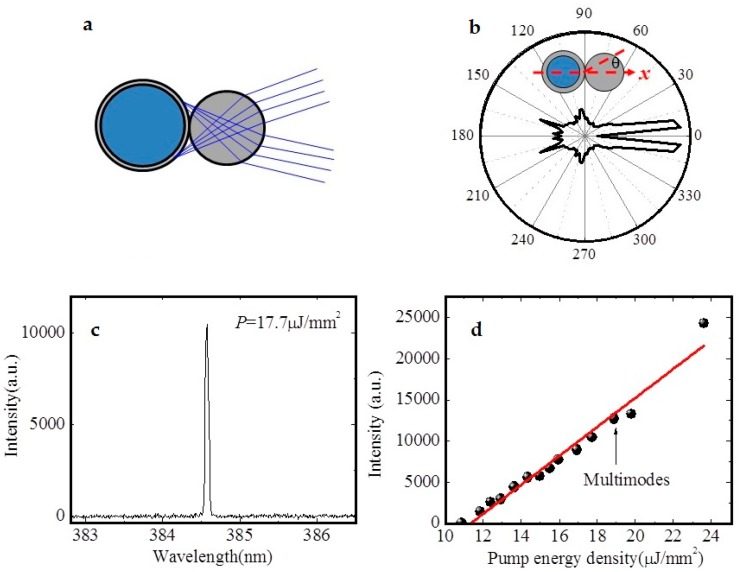
(**a**) Schematic of the focused light rays from the cylinder. (**b**) Far-field distribution of light. (**c**) Single wavelength lasing under lower pump power. (**d**) Plot of the relation between the output intensity and pump energy density [37].

In general, the laser output of the microring resonator lasers is spatially divergent. By the WGM mode-coupling between the lasing resonator and another solid cylinder resonator, the emission direction could be limited to a certain range, thus forming the directional emission. As shown in Figure 5, Tu reported the uses of thin-walled capillary and solid cylinder to construct the coupled ring resonator dye laser [37]. Ultraviolet single-frequency laser emission was generated with a pump threshold of 5.9 μJ·mm^−2^. The laser emission was mainly in two directions with a divergence of 10.5°. The single mode lasing of different power was realized by changing the position of the two resonators.

In another work, Lee [40] used riboflavin water solution as the gain medium to construct the microring optofluidic lasers based on microcapillary tube and optical fiber, respectively. Riboflavin has good biocompatibility as compared to other organic dyes. Multimode lasing at 520–560 nm band was obtained by side pumping of optical parametric oscillator (OPO) laser. The threshold was several tens to one hundred μJ·mm^−2^.

In addition to the liquid gain media, the solid gain layer was also proposed by coating dye-doped polymer on the surface of microcapillary resonators and was first demonstrated by Francois [41]. Usually, the laser features vary with the thickness of gain layer and the solution refractive index in the capillary. The proper thickness range of polymer was 600–800 nm. The multimode lasing of 590–630 nm was generated under the excitation of 532 nm laser by the side pumping. The excitation threshold was lowered to 1.2 μJ (thickness of 800 nm) and 16 μJ (thickness of 600 nm), respectively.

Hollow-core micro-structured optical fibers have a smaller scale than the microcapillaries, and have thus lower internal connection losses. They are often used as miniaturized resonators by tapering. The cavity length is different along the axis. This feature could be applied to frequency tuning. Recently, Liu group [38] proposed a tunable microring dye laser, in which RhB and R6G were used as gain media. The threshold of 16–44 nJ/pulse was obtained by the axial pumping. The tuning range was 10 nm. Besides, Yu [39] constructed a single longitudinal mode optofluidic microring laser by the hollow microstructure fiber. The effective cavity length was about 109.3 µm. The dye fluid was injected into the hollow fiber. The threshold was lowered to 664 nJ·mm^−2^ by the side pumping. Different dyes were used for laser emission of different wavelengths.

**Figure 6:**
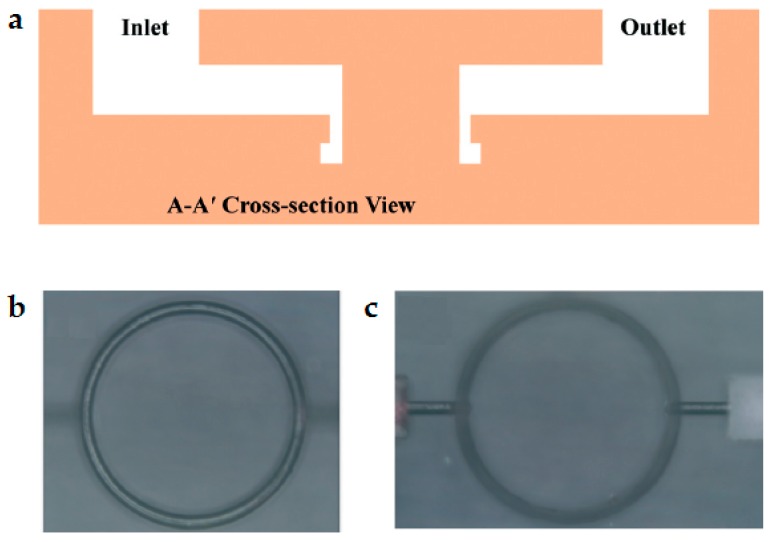
(**a**) Cross-sectional view of the monolithic optofluidic ring resonator; (**b**,**c**) Micrographs of the bottom and the top by focusing on the ring and the fluid delivery channel, respectively [2].

In general, the planar liquid-core microring resonator requires a liquid-core waveguide channel to connect to the liquid microring resonator channel to transport the gain medium, which inevitable results in a decrease of the Q-factor. By using the three-dimensional (3D) direct-writing of the femtosecond laser, the gain medium inlet and outlet channels could be designed in the non-WGM area and the high Q-factor could be maintained using the 3D pipeline design. Monolithic microring laser on glass substrate was first reported by Fan’s group [2]. The ring cavity had the inner radius, the outer radius and depths of 150 µm, 170 µm and 40 µm, respectively. As shown in Figure 6, R6G dye was dissolved in a quinoline solution with refractive index 1.62 to act as the gain medium. It was pumped by nanosecond pulses which were generated by a 532-nm optical parametric oscillator (OPO) laser. Since the fluid refractive index was bigger than the glass, the WGM wave was mainly confined in the fluid close to the outer edge. The lasing spectrum was multimode and the lasing threshold was approximated 15 µJ·mm^−2^.

**Figure 7:**
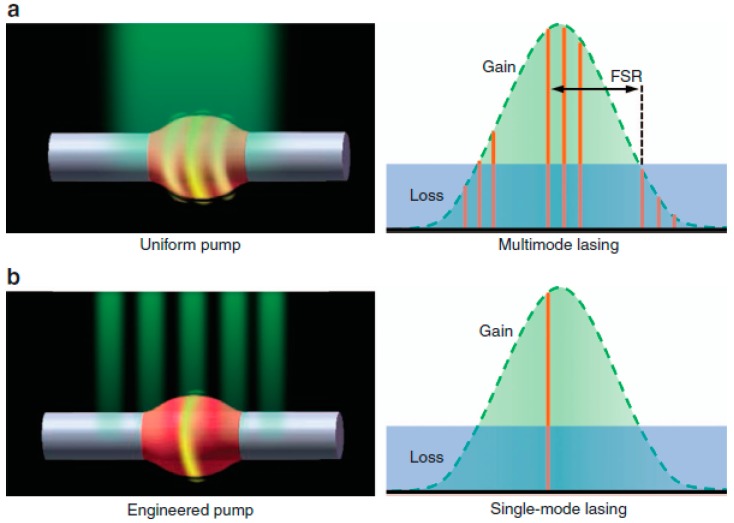
WGM dye laser based on the microbottle structure. (**a**) Uniform pump and multimode lasing; (**b**) modulation pumping and single mode lasing [42].

The WGM dye lasers based on the microbottle have multi-wavelengths distributed along the axis. A single WGM lasing mode could be obtained by the spatial modulation approach of pumping, which may result from the laser-interference excitation field. Gu’s group [42] proposed WGM lasing in dye-doped polymer microbottle resonators, as shown in Figure 7. The pump energy distribution profile along the axis could be rearranged by adjusting the angle between the two excitation beams. The lasing might be single mode by tuning the space of the fringes along the axis and the frequency could be tuned by applying a tensile stress along the fiber axis.

Optofluidic lasers with a single molecular layer of gain was first reported by Fan’s group [48,49]. The gain layers used enhanced green fluorescent protein (eGFP), dye-labeled bovine serum albumin (BSA) and dye-labeled DNA, and were assembled on the surface of ring resonators by the surface immobilization biochemical methods. This is a very interesting work for high sensitivity surface bio-detection.

The immiscible dye droplets suspended in the solution are excellent disk-like optical resonators. They also produce lasing output under proper pumping and can be tuned by the solution interfacial tension. Yang [51] used the inkjet print technology to inject a gain medium solution to float on the water to form a fully liquid WGM microlaser. The tension was changed by the concentration of soap water.

## 3. Optofluidic Microcavities for Biosensors

The physical mechanism of the bio-sensing using the optical microcavities is that the electric field distribution is changed by the variation of refractive index of surrounding medium. The redistribution of electric field would alter the cavities’ resonance mode, which in turn would vary the time (or frequency) domain features of signal light, such as a resonant peak shift, resonant mode splitting, broadening, and intensity variation, etc. By detecting these optical features, the concentration of species can be obtained, which is highly related to the refractive index of sample solution. Especially for the WGM cavities with high Q factor, standing waves are formed around the ring due to the long light travelling time (or distance). Even if a single bio-particle of nanoscale is locally attached to the cavities’ surface, it would also rearrange the electric field distribution because of highly enhanced light-particle interaction. Recently, much research attention has been put on the WGM-based detection of single particles, such as virus, DNA and single proteins [19,20,21,22,23,24,25,63,64,65,66,67,68,69,70,71,72,73,74,75,76,77,78,79]. Some reviews had made detailed descriptions of the theories and the recent biosensing applications of optical microresonators [27,28,67,68]. Here, we will just review the bio-sensing from the two supplementary aspects: microcavity-based active biosensing and microcavity-based passive biosensing.

### 3.1. Microcavity-Based Active Biosensing

When the target concentration is extremely low, the traditional fluorescent intensity-based bio-detecting methods hardly work due to the low signal intensity and various types of noises. For example, enzyme-linked immunosorbent assay (ELISA) kits rely on the intensity of fluorescence generated from the product of the enzyme-substrate reaction so as to quantify the targets attached to the solid surface of ELISA kits. The detection limits are usually sub-μg·L^−1^ for most targets’ bulk solutions and are hard to decrease further due ro the influences of nonspecific bindings, the auto-fluorescence of materials and leakage of excitation light. To improve the detection limit of ELISA, Fan’s group [12] incorporated the PPFP cavities into the ELISA kits, in which fluorescence was confined and resonated to lasing by detecting the lasing onset time to obtain the concentration of interleukin-6 solutions. The threshold of the laser was below 320 μJ·mm^−2^ using the 532-nm OPO pulsed laser pumping. The detection limit was reduced to 1 fg·mL^−1^ and the dynamic range was extended to 10^6^. A similar detection method was applied to the photocatalytic reaction by the same group [13], which constructed an optofluidic catalytic laser for ultra-sensitive sulfide ion detection.

Besides this, the fluorescence resonance energy transfer (FRET) process can be incorporated into the microcavity to form a laser-based sensing platform, which would greatly improve the sensitivity of bio-sensing [15,16]. More descriptions of the theories of FRET laser-based sensing can be also found in [17].

Moreover, Ren [63,64] proposed an optofluidic laser for high-sensitivity and low-detection-limit sensing of refractive index, which obtained the sensitivity of 3874 nm/RIU and the noise equivalent detection limit of 2.6 × 10^−6^ RIU. Zhang [65] improved the refractive index sensitivity of the microring laser by two orders of magnitude via the strong coupling between the ring laser and the fluidic microtube.

### 3.2. Microcavity-Based Passive Biosensing

For the passive bio-sensors based on microcavities, the researchers mainly focused on WGM-based resonators due to the powerful detection abilities of surface bio-reactions. Different configurations of resonators have been implemented for label-free bio-sensing, such as cylindrical ring, bottle, bubble-like, disc or toroidal, and planar liquid core ring [19,20,21,22,23,24,25,26]. As the passive sensors, no gain medium is needed and thus no fluorescence or lasing is produced. An external light source (white source or tunable laser) is used to couple photons into the cavities by the taper fiber or the waveguide. By monitoring the shift or splitting the resonant peaks of transmission spectra, analyte concentration or molecule attaching can be detected [69,70,71,72,73,74,75,76,77,78,79,80,81,82,83,84]. As the optical confinement elements, the cavities with high Q factor would greatly enhance the light-matter interaction and would result in high sensitivity. However, there are some potential problems such as light source fluctuation, temperature variation, large background caused by the low couple efficiency of excitation, detector noises, etc. All of these factors would deteriorate the detection limit significantly.

Hybrid microcavities have been reported to utilize the plasmon resonances to further enhance the light–matter interaction [85,86,87,88,89]. Advanced signal processing techniques such as self-reference differential detection, frequency locking (or phase locking detection) are developed to improve the signal and noise ratio (SNR) [90,91,92,93,94,95,96,97,98]. The detection limit is reduced to the level of ~5 kDa for a single bio-particle. An excellent example of this kind of work was reported by Zhang [91], who developed a self-referenced differential mode sensing method. It used two resonant modes in the same microbottle resonator to reduce the measurement noises from the exciting source fluctuation. The detection limit of 10 fg·mL^−1^ for bovine serum albumin molecules was obtained. Su [94] used the laser frequency locking technique to improve the SNR of microtoroid resonators, obtaining the detection of single nanoparticles of 2.5 nm in radius and 15.5 kDa molecular weight. In addition to the detection of liquid concentration and single particles, these sensors could also be used for gas sensing applications [99,100].

**Figure 8:**
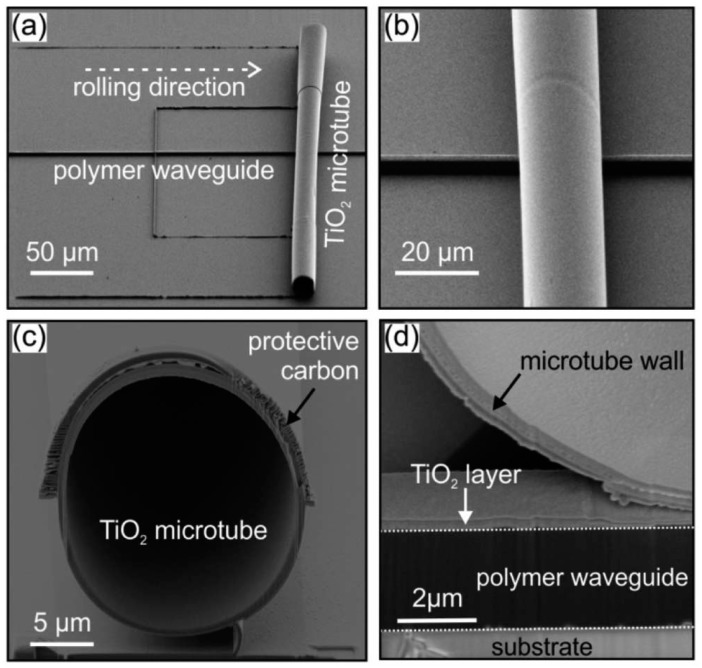
Lab-in-a-tube system made of rolled-up TiO_2_ microresonators integrated with polymer waveguides. (**a**) Microtube was rolled-up by a U-shaped pattern. (**b**) Close-up view of the tube that is connected tightly with the polymer waveguide to ensure optimal optical coupling. (**c**) Compact winding layers of the tube wall were revealed by a FIB cut with the protection of a carbon layer. (**d**) The FIB cut image at the waveguide revealed the compact tube wall in the vicinity of the polymer waveguide [101].

Although optofluidic microcavities based on WGM have great potential in sensing, the integration on a single chip remains a big challenge. For example, high-quality 3D microdisk (or microtoroid) resonators could be easily fabricated by laser processing on glass or silicon substrates, but the photonic waveguides that are necessary for delivering the probe light are hard to fabricate. Taper fibers are usually applied and more extra processing is needed. Yet the uncertainty regarding the geometric parameters of taper fiber and the gap between them are severe hindrances to bulk production. Resonators made by microcapillaries or hollow fibers are also difficult to integrate on a monolithic chip owing to their large sizes and structural fragility. Recently, Schmidt’s group [101] has developed an innovative approach called *lab-in-a-tube*, which integrates numerous rolled-up components into a single device on a chip. Figure 8 shows a TiO_2_ microtubular optical resonator as a result of the rolling-up of a 2D planar membrane deposited on the substrates due to the surface stress. In addition, the resonator is integrated with vertical-sited SU-8 polymer waveguide. The geometric parameters of microtube, the waveguide and the gap between them were well defined and controllable at nanoscale. The test results showed that the resonators had good sensing performance and excellent optical coupling efficiency with an extinction ratio of 32 dB over the communication band. Other materials such as SiO_2_ was also developed by the same group [102,103]. These studies made an important contribution to the research of optofluidic monolithic integration.

## 4. Conclusions

Optofluidic microcavities have found wide applications and are still expanding their application areas rapidly. Here, we have summarized the recent progress in the areas of microlasers and biosensors. Generally, the optofluidic microlasers are developing toward high Q factors with a low threshold, small volume, easy mode controllability and wide tunability. In addition, the high performance of microresonators improves the light-matter interaction and thus greatly enhances the sensing abilities and the scope of applications. By means of process improvement, structure integration and detection method innovation, new microcavity devices with higher performance are presented continuously. It is expected that more practical devices will be developed for lasers, biosensors and other applications.

## Figures and Tables

**Table 1:** List of the optofluidic lasers based on the Fabry–Pérot microcavities.

Ref.	Cavity Configuration	Cavity Length (μm)	Q-Factor	Threshold (μJ·mm^−2^)	Lasing Mode	Gain Materials	Cavity Materials
[29]	PCFP	31	5.6 × 10^5^	0.09	Mutlimode	R6G	Fused Silica substrate
		8	5.6 × 10^5^	0.7	Single mode	R6G	
[30]	PCFP	39	4 × 10^5^	0.13	Mutlimode	R6G	Fused Silica substrate
[31]	PPFP	150		9.6	Mutlimode	MB	Fused Silica plate
[32]	PPFP	165		1.3	Mutlimode	IgG-Atto488 complex	Fused Silica plate
[33]	PPFP	~10,000		1	Mutlimode	Rh640	Fiber, caplilary

**Table 2:** List of the optofluidic lasers based on the WGM microcavities.

Ref.	Cavity Configuration	Cavity Length (μm)	Q-Factor	Threshold	Lasing Mode	Gain Materials	Cavity Materials
[37]	Cylindrical ring resonator	~410	2.6 × 10^6^	5.9 μJ/mm^2^	Single mode, 386.75 nm	LD390	Microcapillary, glass solid cylinder
[38]	Cylindrical ring resonator	59.9–90.9		16–44 nJ/pulse	~10 nm tunable range, axial pumping	R6G, RhB	Hollow core microstructured fiber
[39]	Cylindrical ring resonator	17.4		664 nJ·mm^−2^	Single longitudinal mode, lateral pumping	R6G	Hollow core microstructured fiber
[40]	Cylindrical ring resonator	157,393		Several tens μJ/mm^2^	Mutlimode, 520–560 nm	Ribo-flavin	Microcapillary
[41]	Cylindrical ring resonator	157	6000	1.2 μJ	Mutlimode, 600–615 nm	Nile red dye	Microcapillary, polymer
[48]	Cylindrical ring resonator	393	~106	23 μJ/mm^2^	Mutlimode, 510–520 nm	eGFP	Bare SM-28 fiber
[2]	Monolithic liquid-core ring resonator	534	3.3 × 104	15 μJ/mm^2^	Mutlimode, 570–580 nm	R6G	Glass
[42]	Microbottle	9–19		10–20 μW/mm^2^	Single mode, 580–620 nm, tunable	R6G	Microfiber, polymer
[4]	Microbottle	534		~3.6 mW	Multimode, 1530–1540 nm	Er: Yb doped glass	glass
[51]	Droplet	323	5800		Multimode, 590–610 nm	R6G	Dichloro-methane and epoxy resin
